# Planned anatomy-guided dual-sided (“Sandwich”) ablation improves recurrence-free survival in idiopathic premature ventricular contractions: a propensity-matched cohort study

**DOI:** 10.1007/s10840-026-02252-7

**Published:** 2026-02-19

**Authors:** Serkan Topaloglu, Ahmet Korkmaz, Elif Hande, Ozcan Cetin, Salih Can Özdoğan, Kürşat Kağan Yenikomşu, Duygu Kocyigit, Meryem Kara, Ozcan Ozeke, Dursun Aras, Tolga Aksu, Serkan Cay, Fırat Ozcan

**Affiliations:** 1 Cardiology Department, University of Health Sciences, Ankara Bilkent City Hospital, Bilkent, Ankara, Turkey; 2 Cardiology Department, Istanbul Medipol University, Bahçelievler, İstanbul, Turkey; 3 MedicalPark Florya Hospital, Cardiology Department, Istinye University, İstanbul, Turkey

**Keywords:** Ventricular tachycardia, Catheter ablation, Premature ventricular contraction, Radiofrequency, Intramural

## Abstract

**Purpose:**

Catheter ablation of idiopathic premature ventricular contractions (IPVCs) is often challenged by intramural or anatomically complex substrates, where targeting only the site of earliest activation may result in insufficient lesion transmurality and arrhythmia recurrence. We aimed to determine whether a planned, anatomy-guided dual-sided (“sandwich”) ablation strategy improves long-term clinical outcomes compared with conventional ablation.

**Methods:**

We conducted a retrospective cohort study of patients undergoing catheter ablation for IPVCs. After exclusions, 412 patients were eligible, and 1:1 propensity score matching based on left ventricular ejection fraction, QRS duration, PVC burden, and anatomical localization yielded 97 well-balanced pairs (*n* = 194). The planned sandwich ablation strategy consisted of conventional ablation at the earliest activation site followed by systematic radiofrequency applications from anatomically adjacent or opposing myocardial surfaces. The primary endpoint was recurrence-free survival, defined as absence of sustained ventricular arrhythmia or PVC burden > 1% during follow-up; major complications constituted secondary endpoints.

**Results:**

Among 194 propensity-matched patients (mean age 50.6 ± 11.3 years; 52.1% male), sandwich ablation was associated with significantly higher recurrence-free survival compared with conventional ablation (94.8% vs. 78.4%; *p* = 0.001). Subgroup analysis demonstrated a marked benefit of sandwich ablation in patients with earliest activation time > − 30 ms (92.5% vs. 60.5%; *p* < 0.001). On multivariate Cox regression analysis, sandwich ablation emerged as an independent predictor of reduced recurrence (adjusted hazard ratio 0.28; 95% CI 0.10–0.76; *p* = 0.013). Major complication rates were comparable between groups.

**Conclusions:**

Planned dual-sided ablation may improve recurrence-free survival in IPVCs.

**Graphical Abstract:**

**Figure:**
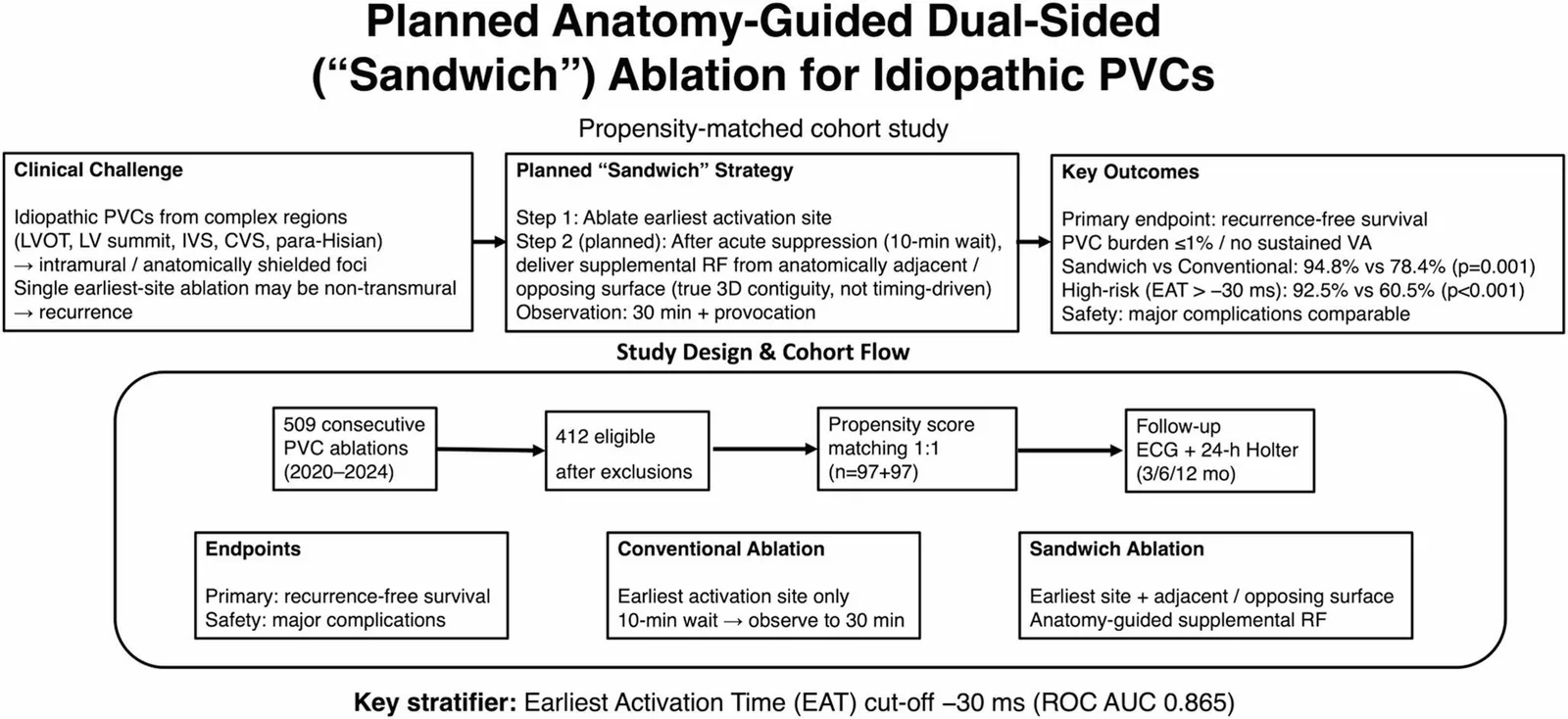

**Supplementary Information:**

The online version contains supplementary material available at https://doi.org/10.1007/s10840-026-02252-7.

## Introduction

Catheter ablation represents the primary interventional therapy for ventricular arrhythmias (VAs); nevertheless, achieving durable success remains challenging, particularly when arrhythmias originate from anatomically complex regions—such as the left ventricular outflow tract (LVOT), interventricular septum (IVS), or the coronary venous system—or when the arrhythmogenic substrate is deeply intramural [1, 2]. The three-dimensional architecture of the ventricular myocardium, combined with preferential conduction pathways and limited catheter accessibility, often obscures the true site of origin and hinders the creation of effective transmural lesions, ultimately leading to incomplete ablation and procedural failure [3–5].

To address these challenges, several advanced techniques—such as prolonged radiofrequency (RF) delivery, adding additional ablation sets, bipolar ablation, and needle-based intramural ablation—have been developed [6–9]. Although these approaches offer promise in selected cases, they are often reserved for bailout scenarios after the failure of standard endocardial ablation and are associated with increased procedural complexity, prolonged operative times, and elevated complication rates [10, 11].

Recognizing the limitations of single-site ablation, recent anatomical and electrophysiological studies have emphasized the potential benefit of targeting anatomically adjacent surfaces to the site of earliest activation [11, 12]. Sequential or dual-sided ablation strategies, by leveraging adjacent anatomical vantage points, aim to achieve deeper, more transmural lesions and thereby improve long-term arrhythmia-free survival. This approach appears particularly relevant in challenging regions such as the left ventricular summit (LVS), IVS, and basal septal areas, where arrhythmic foci may exhibit intramural extensions or epicardial components [5, 13, 14].

While previous applications of dual-sided or anatomically guided ablation have largely been reactive—employed after the failure of initial single-site attempts—the efficacy and safety of a planned, systematic anatomy-guided dual-sided ablation strategy across a broader range of anatomically complex VAs has not been well established [15].

In this study, we aimed to evaluate the efficacy and safety of a systematic ablation strategy that involved targeting not only the site of earliest activation but also its anatomically adjacent regions, in order to enhance lesion transmurality, procedural success, and long-term arrhythmia-free survival in patients with ventricular arrhythmias.

## Methods

### Study design and patient population

This single-center, retrospective observational cohort study included a total of 509 consecutive patients who underwent catheter ablation for idiopathic premature ventricular complexes (PVCs) at our institution between January 2020 and December 2024. The study protocol was approved by the local institutional review board and was conducted in accordance with the principles of the Declaration of Helsinki.

Eligible patients met the following criteria: undergoing catheter ablation targeting a single, monomorphic PVC focus; availability of comprehensive electroanatomic mapping and activation timing data; and a minimum follow-up duration of 6 months post-ablation.

## Group assignment

Patients were considered eligible for group assignment when acute elimination of PVCs was observed within the first 10-minute waiting period following ablation at the earliest activation site. In our electrophysiology laboratory, a standardized waiting-period protocol is routinely applied in all PVC ablation procedures. Accordingly, all patients underwent an initial 10-minute observation period after acute PVC elimination.

After confirmation of acute success during this initial 10-minute waiting period, the subsequent procedural strategy was determined according to operator-specific routine practice. In operators who routinely perform a planned sandwich ablation strategy, additional anatomically guided lesions were systematically delivered. In contrast, operators favoring the conventional approach did not deliver further lesions and continued with observation only.

Importantly, a total observation period of 30 min was uniformly applied in all cases to confirm sustained PVC suppression, independent of the ablation strategy.

Patients were subsequently categorized into two groups based on the ablation strategy:

## Conventional ablation group

After confirmation of acute success during the initial 10 min, no additional lesions were delivered. The procedure was continued with extended monitoring up to 30 min to verify sustained elimination. In the absence of PVC recurrence, the procedure was concluded without further intervention. No additional ablation lesions were delivered from anatomically adjacent or contralateral myocardial surfaces.

## Sandwich ablation group

After acute elimination was documented within the first 10 min, supplementary lesions were systematically delivered from anatomically adjacent or contralateral myocardial regions (e.g., the right ventricular outflow tract (RVOT) –LVOT septal pairs, aortic cusps, coronary venous system, para-Hisian or subvalvular septal regions). Following these applications, an additional 20-minute monitoring period was completed, for a total of 30 min of observation.

## Exclusion criteria

Patients in whom acute elimination of PVCs was not achieved within the initial 10-minute observation, or in whom recurrence occurred during the subsequent 30-minute monitoring period, were excluded from the analysis. In addition, patients with a history of prior PVC ablation, incomplete procedural data, requirement for bipolar or alcohol ablation during the index procedure, or significant structural heart disease that could confound outcomes were also excluded.

### Propensity score matching (PSM)

To minimize selection bias and ensure comparability between treatment groups, 1:1 propensity score matching (PSM) was performed. Propensity scores were estimated using logistic regression based on key baseline variables: left ventricular ejection fraction (LVEF), QRS duration, PVC burden, and anatomical localization of the ectopic focus. Patients were matched using the nearest-neighbor method without replacement, with a caliper width of 0.2 standard deviations of the logit of the propensity score. After matching, 97 well-balanced pairs were generated, resulting in two groups of 97 patients each (sandwich vs. conventional ablation). Covariate balance was assessed using standardized mean differences, with all variables demonstrating SMD < 0.1 after matching, indicating good balance between groups. This process yielded two well-balanced groups suitable for comparative analysis. Operator identity and experience were not incorporated into the matching model and may represent residual confounding.

## Electrophysiological study and ablation protocol

All procedures were performed in a tertiary high-volume electrophysiology laboratory. Antiarrhythmic medications were discontinued for at least five half-lives before the procedure. Three-dimensional electroanatomical mapping was performed using the CARTO 3 system (Biosense Webster, Diamond Bar, CA, USA), guided by activation and pace mapping strategies. An irrigated, contact force-sensing ablation catheter (Thermocool SmartTouch, Biosense Webster) was utilized in all cases.

Based on electroanatomical mapping, the earliest activation site was identified as the ablation target. Earliest activation time (EAT) was defined as the interval from the onset of the local ventricular electrogram at the mapping site to the onset of the QRS complex on the surface ECG, expressed in milliseconds. A typical site was considered suitable for ablation if it met the following criteria: [1] local activation preceded at least 20 ms from the onset of the surface QRS complex, and [2] a QS morphology was present in the unipolar electrogram. Additionally, a QS pattern in the bipolar electrogram was sought whenever possible.

Following ablation, acute procedural success was verified using a standardized protocol that included a predefined waiting period and arrhythmia provocation with isoproterenol infusion (2–4 µg/kg/min). Programmed ventricular stimulation was used adjunctively when appropriate, recognizing that PVC mechanisms may include automaticity or triggered activity. In the absence of spontaneous or inducible PVC recurrence, the procedure was considered acutely successful.

In the sandwich ablation group, after acute elimination of PVCs was achieved at the earliest activation site, additional radiofrequency applications were delivered to anatomically adjacent or opposing myocardial regions based on true three-dimensional contiguity with the primary site of earliest activation. Septal RVOT and LVOT origins were complemented by ablation at the opposing outflow tract septum. PVCs arising from the right coronary cusp were complemented by ablation at the contiguous septal or subpulmonic RVOT, whereas left coronary cusp origins were complemented by ablation at the subvalvular LVOT and aortomitral continuity. Left ventricular summit origins were approached using adjacent endocardial and coronary venous or aortic root vantage points. Parahisian origins were complemented by ablation at adjacent septal surfaces and, when appropriate, at the right or non-coronary aortic cusps, whereas basal inferoseptal (crux) origins were complemented by ablation within the proximal coronary sinus or middle cardiac vein. Supplemantary Table 1. The selection of sandwich targets was guided by anatomical proximity rather than earliest activation timing at the secondary site Lesion delivery was adjusted according to the presumed depth and accessibility of the arrhythmogenic substrate, aiming to achieve lesion sets from opposing surfaces when feasible. Although the sandwich technique involves sequential unipolar radiofrequency applications, it represents a planned anatomy-guided strategy rather than a bailout or reactive approach, with the aim of targeting deep or intramural substrates.

Coronary angiography was systematically performed prior to ablation attempts near the coronary venous system or aortic root to ensure a safe distance (> 5 mm) from major coronary arteries. Lesions were not delivered within venous structures if this distance could not be assured.

Radiofrequency (RF) energy settings were standardized as follows: 30–40 W for standard endocardial lesions, 20–30 W for venous or epicardial structures, 25–35 W for lesions delivered within the aortic root or aortomitral continuity region. Irrigation flow was maintained at 17–25 mL/min. Successful RF application was defined by real-time impedance drop (> 10 ohms), local electrogram attenuation, or loss of capture during pacing at 10 mA and 2 ms. Importantly, in the sandwich group, this two-site strategy was applied as a predefined procedural protocol, not as a bailout technique.

## Cut-off definition and subgroup stratification

To determine a clinically meaningful threshold of pre-QRS activation time predictive of PVC recurrence, a receiver operating characteristic (ROC) curve analysis was performed. The optimal cut-off value was identified using the Youden index. Based on this threshold, patients were stratified into two subgroups: the high-risk group (activation time ≤ cut-off) and the low-risk group (activation time > cut-off). Subsequently, the comparative effectiveness of sandwich versus conventional ablation was specifically evaluated within the high-risk subgroup to assess their differential impact on long-term clinical outcomes.

### Follow-up protocol and clinical outcomes

Post-ablation follow-up included scheduled visits at 3, 6, and 12 months, with 12-lead ECG and 24-hour Holter monitoring. Additional assessments were performed in the presence of recurrence-related symptoms.

The primary endpoint was arrhythmia-free survival, assessed by 12-lead electrocardiogram and 24-hour Holter monitoring at 3, 6, and 12 months. Arrhythmia recurrence was defined as documented sustained VA or a PVC burden exceeding 1% on follow-up Holter studies.

Safety endpoints were defined as major procedure-related complications, including pericardial tamponade, coronary artery injury, steam pops, and high-grade atrioventricular (AV) conduction block. All adverse events were documented in accordance with standard reporting criteria and evaluated by an independent investigator blinded to group allocation.

### Statistical analysis

Continuous variables were expressed as mean ± standard deviation and compared using the independent samples t-test or Mann-Whitney U test, as appropriate based on distribution. Categorical variables were presented as frequencies and percentages, and compared using the chi-square test or Fisher’s exact test.

To assess covariate balance before and after propensity score matching, standardized mean differences (SMDs) were calculated for all baseline variables. As shown in Supplemantary Table 2, all covariates exhibited SMDs < 0.1 after matching, indicating good balance between the sandwich and conventional ablation groups. This suggests that the matched cohorts were well balanced in terms of demographics, PVC burden, and electrocardiographic characteristics, thereby minimizing the risk of confounding in subsequent comparative analyses.

Univariate and multivariate Cox proportional hazards regression analyses were conducted to identify independent predictors of PVC recurrence. Variables with a p-value < 0.10 in univariate analysis were included in the multivariate model. Hazard ratios (HRs) and 95% confidence intervals (CIs) were reported. The proportional hazards assumption was verified using Schoenfeld residuals.

Receiver operating characteristic (ROC) curve analysis was used to determine the optimal cut-off value for pre-QRS activation time in predicting recurrence. The area under the curve (AUC), sensitivity, specificity, and 95% CI were calculated. Based on the ROC-derived threshold (–30 ms), patients were stratified into two risk groups: high-risk (activation time < − 30 ms) and low-risk (activation time ≥ − 30 ms).

Survival curves were estimated using the Kaplan-Meier method, and differences between groups were assessed with the log-rank (Mantel-Cox) test. All statistical tests were two-tailed, and a p-value < 0.05 was considered statistically significant. Analyses were performed using SPSS version 26.0 (IBM Corp., Armonk, NY, USA) and R version 4.2.2 (R Foundation for Statistical Computing, Vienna, Austria).

## Results

A total of 509 patients who underwent catheter ablation for idiopathic PVCs between January 2019 and March 2025 were initially screened. After excluding patients with missing follow-up data or incomplete procedural information, 412 patients were eligible for analysis. Among these, 97 patients received sandwich ablation, while the remaining 315 underwent conventional ablation. Using 1:1 propensity score matching based on LVEF, QRS duration, PVC burden, and anatomical localization, 97 matched pairs were created, resulting in two well-balanced groups of 97 patients each.

### Baseline characteristics

A total of 194 patients were included after 1:1 propensity score matching, with 97 patients in each group (sandwich vs. conventional ablation). The mean age of the study population was 50.6 ± 11.3 years, and 52.1% were male. There were no significant differences between groups in terms of hypertension, diabetes mellitus, left ventricular ejection fraction (LVEF), left atrial diameter, or PVC burden (all *p* > 0.05) (Table 1).

**Table 1:** Baseline and procedural characteristics of study groups

Variable	Sandwich ablation (*n* = 97 patients)	Conventional ablation (*n* = 97 patients)	*p*-value
Baseline Characteristics			
Age (years), mean ± SD	49.8 ± 11.0	51.4 ± 11.5	0.28
Male sex, n (%)	53 (54.6%)	48 (49.5%)	0.47
Hypertension, n (%)	20 (20.6%)	23 (23.7%)	0.60
Diabetes mellitus, n (%)	9 (9.3%)	11 (11.3%)	0.64
LVEF (%), mean ± SD	57.3 ± 6.2	56.8 ± 6.8	0.46
LVEDD (mm), mean ± SD	47.9 ± 3.3	48.1 ± 3.1	0.59
LA diameter (mm), mean ± SD	36.7 ± 5.3	37.3 ± 5.5	0.38
PVC burden (%), mean ± SD	20.4 ± 8.9	19.8 ± 9.3	0.61
Procedural Characteristics			
Total procedural time, min (mean ± SD)	141.1 ± 40.2	120.8 ± 38.1	0.01
RF application time, min (mean ± SD)	20.5 ± 10.7	14.1 ± 9.8	0.003
Number of lesions delivered, mean ± SD	15.8 ± 4.3	9.6 ± 3.8	< 0.001
RVOT, n (%)	25 (25.8%)	28 (28.9%)	0.61
LVOT, n (%)	60 (61.9%)	56 (57.7%)	0.51
• Aortic cusps (LCC/RCC/NCC), n (%)	22 (22.7%)	21 (21.6%)	1.000
• LV summit, CVS, n (%)	24 (24.7%)	23 (23.7%)	1.000
• Subvalvular/Subaortic, AMC n (%)	14 (14.4%)	12 (12.4%)	0.833
Others (Parahisian, Crux), n (%)	12 (12.4%)	13 (13.4%)	1.000
Anatomical Adjacent Ablation Performed, n (%)	97 (100%)		
• Coronary sinus (GCV/AIV), n (%)	30 (30.9%)		
• Septal RVOT, n (%)	31 (32.0%)		
• Subvalvular LVOT, n (%)	22 (22.7%)		
• Others (Parahisian, Crux), n (%)	14 (14.4%)		

Abbreviations: AIV = anterior interventricular vein; AMC = aortomitral continuity; Crux = atrioventricular crux region; GCV = great cardiac vein; LA = left atrium; LCC = left coronary cusp; LV = left ventricle; LVEDD = left ventricular end-diastolic diameter; LVEF = left ventricular ejection fraction; LVOT = left ventricular outflow tract; NCC = non-coronary cusp; PVC = premature ventricular complex; RCC = right coronary cusp; RF = radiofrequency; RVOT = right ventricular outflow tract; SD = standard deviation

The total procedural time was significantly longer in the sandwich group compared to the conventional group (141.1 ± 40.2 vs. 120.8 ± 38.1 min, *p* = 0.01). Similarly, RF application time (20.5 ± 10.7 vs. 14.1 ± 9.8 min, *p* = 0.003) and the number of lesions delivered (15.8 ± 4.3 vs. 9.6 ± 3.8, *p* < 0.001) were significantly higher in the sandwich group. Anatomical distribution of earliest activation sites was similar between groups (*p* > 0.05 for all), including RVOT, LVOT (aortic cusps, LVS, subvalvular), and other sites (Table 1).

### Freedom from PVC recurrence

During follow-up, the sandwich ablation group demonstrated significantly higher freedom from PVC recurrence compared to the conventional group (94.8% vs. 78.4%, *p* = 0.001). In subgroup analysis by anatomic site, this effect was statistically significant for LVOT PVCs (*p* = 0.017), particularly those arising from the LVS (*p* = 0.036). Although RVOT outcomes numerically favored the sandwich approach (100% vs. 82.1%), this difference did not reach statistical significance (*p* = 0.052) (Table 2).

### Safety outcomes

The overall incidence of procedural complications was low and comparable between the two groups. Major complications occurred in 3.1% of patients in both the sandwich and conventional ablation groups. There was one case of pericardial tamponade in each group, while no coronary artery injuries, high-grade atrioventricular blocks, or procedure-related deaths were reported. Steam pops were observed in 1 patient (1.0%) in the sandwich group and in 2 patients (2.1%) in the conventional group (*p* = 0.56). Minor vascular complications were infrequent and not statistically different between groups (*p* = 0.65) (Table 2).

**Table 2:** Primary and safety outcomes of study groups

Primary Outcomes- Freedom from PVC			
Region / Earliest Site	**Sandwich** **Ablation Group**	**Conventional** **Ablation Group**	***p*** **-value**
RVOT	100.0% (25/25)	82.1% (23/28)	0.052
LVOT (total)	93.3% (56/60)	76.8% (43/56)	0.017
•Aortic cusps	95.5% (21/22)	85.7% (18/21)	0.344
• LV summit,	91.7% (22/24)	65.2% (15/23)	0.036
• Subvalvular LVOT	92.9% (13/14)	83.3% (10/12)	0.583
Others (Parahisian, Crux)	91.7% (11/12)	76.9% (10/13)	0.593
Total	94.8% (92/97)	78.4% (76/97)	0.001
Safety Outcomes- Complications			
Complication	**Sandwich** **Ablation Group**	**Conventional Ablation Group**	***p*** **-value**
Pericardial tamponade	1 (1.0%)	1 (1.0%)	1.0
Coronary artery injury	0 (0.0%)	0 (0.0%)	1.0
Steam pop	2 (2.1%)	1 (1.0%)	1.0
High-grade AV block	0 (0.0%)	0 (0.0%)	1.0
Vascular complication	3 (3.1%)	2 (2.1%)	1.0
Procedure-related death	0 (0.0%)	0 (0.0%)	1.0
Total complications	5 (5.1%)	3 (3.1%)	0.718

PVC = premature ventricular complex. Freedom from PVC was defined as absence of clinical recurrence or burden > 1% at 6-month follow-up based on ECG or Holter monitoring

### Predictors of recurrence

In univariate Cox regression analysis, several factors were significantly associated with PVC recurrence. Sandwich ablation was linked to a lower risk of recurrence (HR: 0.32; 95% CI: 0.13–0.78; *p* = 0.012), while higher PVC burden (> 24%), longer ablation duration (per 10 s), earliest activation time per 10 ms, RVOT origin, and reduced LVEF (< 50%) also showed significant associations.

In the multivariate model, sandwich ablation remained an independent protective factor (adjusted HR: 0.28; 95% CI: 0.10–0.76; *p* = 0.013). Activation time ≤ − 30 ms (adjusted HR: 2.41; 95% CI: 1.08–5.39; *p* = 0.031), longer ablation duration (adjusted HR: 1.18 per 10 s; 95% CI: 1.03–1.36; *p* = 0.021), and LVEF < 50% (adjusted HR: 2.36; 95% CI: 1.11–5.02; *p* = 0.027) were independently associated with higher recurrence risk. RVOT localization was protective (adjusted HR: 0.38; 95% CI: 0.16–0.88; *p* = 0.024), while PVC burden > 24% did not retain significance in multivariate analysis (Table 3).

**Table 3:** Univariate and multivariate Cox regression analysis

Variable	HR (95% CI)	*p*-value	Adjusted HR (95% CI)	*p*-value
Ablation type (Sandwich)	0.32 (0.13–0.78)	0.012	0.28 (0.10–0.76)	0.013
PVC burden > 24%	2.15 (1.10–4.22)	0.026	1.59 (0.83–3.04)	0.165
Ablation duration (per 10 s)	1.21 (1.05–1.38)	0.008	1.18 (1.03–1.36)	0.021
Earliest Activation time (per 10 ms)	2.83 (1.30–6.17)	0.009	2.41 (1.08–5.39)	0.031
Anatomical localization (RVOT vs. Others)	0.41 (0.18–0.92)	0.031	0.38 (0.16–0.88)	0.024
LVEF < 50%	2.47 (1.01–6.05)	0.049	2.36 (1.11–5.02)	0.027

ROC AUC (multivariate model): 0.821, Hosmer-Lemeshow test: *p* = 0.42

Abbreviatons: LVEF: Left ventricular ejection fraction, RVOT: Right Ventricular Outflow Tract

The model demonstrated good predictive performance with an AUC of 0.821 and satisfactory calibration (Hosmer-Lemeshow *p* = 0.42).

### Activation timing cut-off analysis and risk stratification

**Fig. 1:**
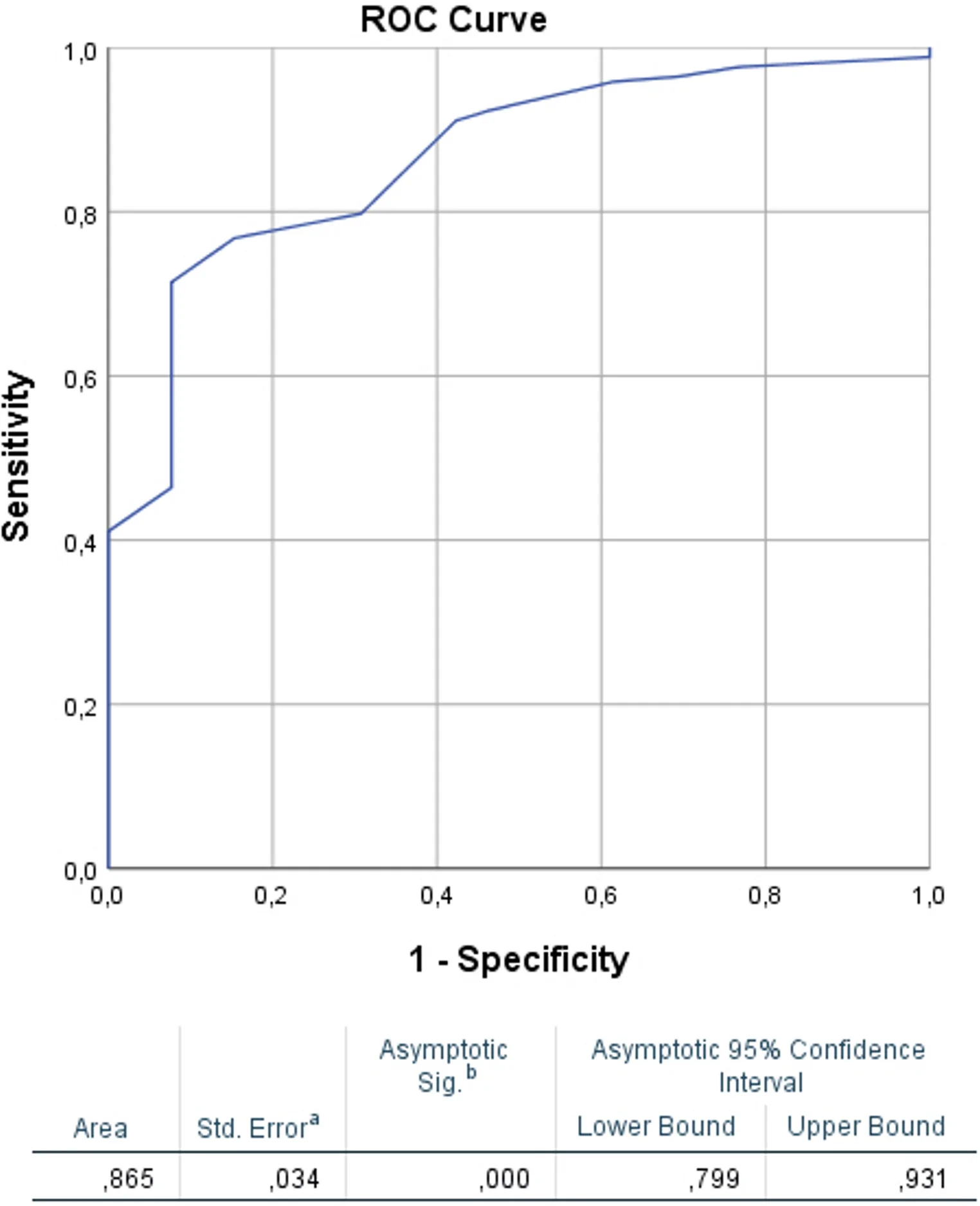
ROC Curve Analysis for Pre-QRS Activation Time Predicting PVC Recurrence. Receiver operating characteristic (ROC) curve evaluating the predictive value of pre-QRS activation time for recurrence of PVCs after ablation. The area under the curve (AUC) was 0.865 (95% CI, 0.799–0.931; *p* < 0.001), identifying − 30 ms as the optimal cut-off. Sensitivity and specificity were calculated using the Youden index

The mean EAT across the cohort was − 31.7 ± 12.9 ms. ROC curve analysis identified a threshold of − 30 ms as the optimal cut-off for predicting PVC recurrence. The AUC was 0.865 (95% CI: 0.799–0.931), with a standard error of 0.034 and *p* < 0.001 (Fig. 1).

This cut-off demonstrated acceptable discriminative power and was subsequently used to stratify patients into two risk categories: a high-risk group (activation time > − 30 ms) and a low-risk group (≤ − 30 ms) (Supplemantary Table 3).

### Recurrence outcomes and subgroup comparison

**Fig. 2:**
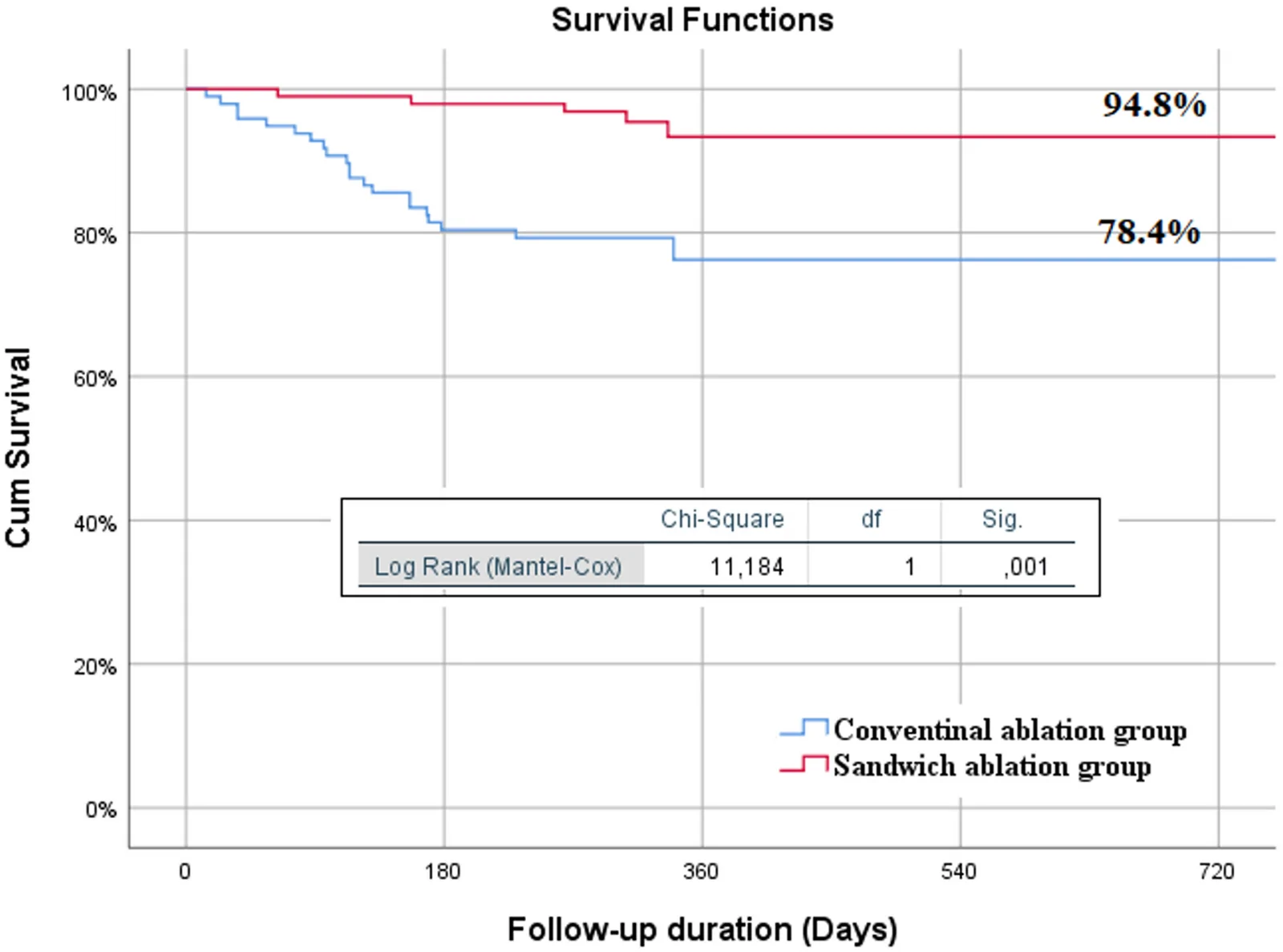
Kaplan-Meier analysis of recurrence-free survival in total study population. Kaplan-Meier curves comparing recurrence-free survival between patients treated with sandwich ablation and conventional ablation for idiopathic premature ventricular contractions (PVCs). Sandwich ablation resulted in significantly higher recurrence-free survival (94.8%) compared to conventional ablation (78.4%) during follow-up (log-rank *p* = 0.001; chi-square = 11.184). EAT = earliest activation time; PVC = premature ventricular contraction

Kaplan-Meier survival analysis demonstrated a significantly higher recurrence-free survival in the sandwich ablation group compared to the conventional group (94.8% vs. 78.4%, log-rank *p* = 0.001; Fig. 2).

**Fig. 3:**
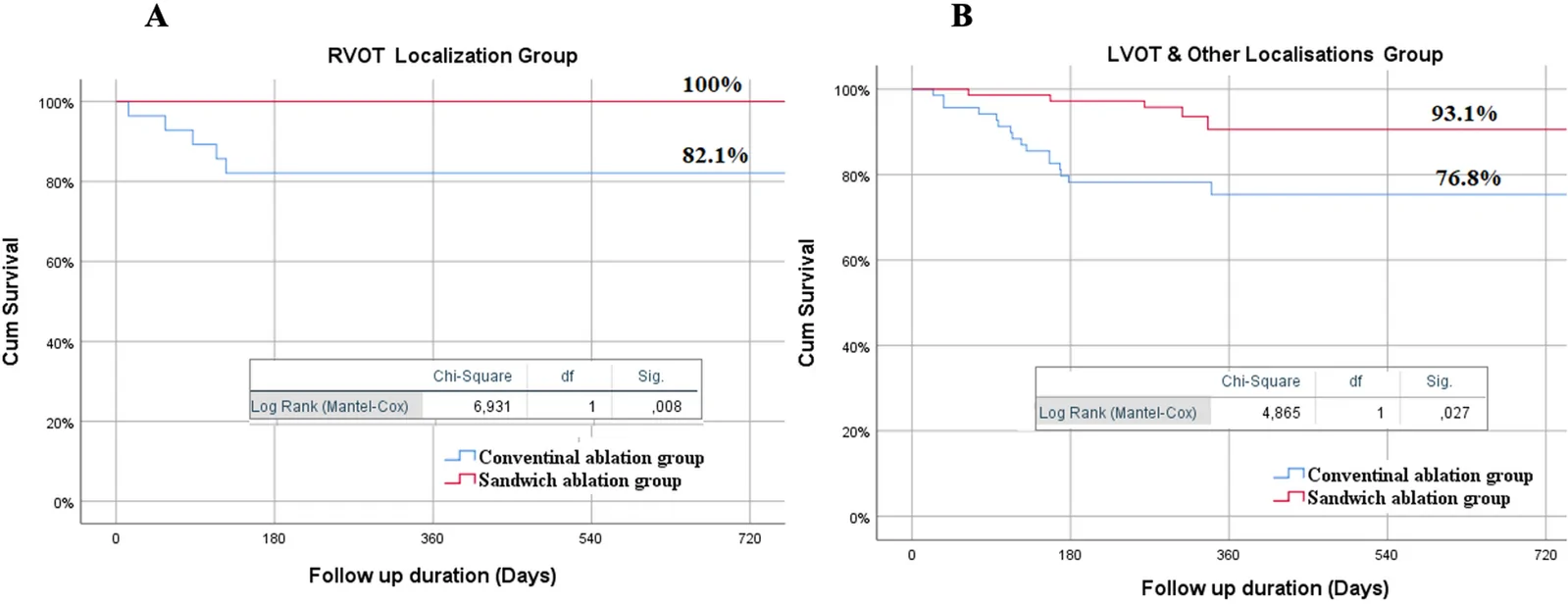
Recurrence-free survival stratified by anatomical PVC Origin. Kaplan-Meier curves showing recurrence-free survival by PVC origin. In RVOT-origin PVCs, sandwich ablation was associated with 100% recurrence-free survival vs. 82.1% in the conventional group (*p* = 0.008). In LVOT & other localizations, recurrence-free survival was 93.1% vs. 76.8%, respectively (*p* = 0.027). RVOT = right ventricular outflow tract; LVOT = left ventricular outflow tract

When stratified by PVC origin, sandwich ablation consistently showed superior outcomes. In patients with RVOT-origin PVCs, recurrence-free survival was 100% in the sandwich group compared to 82.1% in the conventional group (log-rank *p* = 0.008; Fig. 3A). Similarly, among those with PVCs arising from LVOT or other non-RVOT sites, the sandwich group had a significantly higher survival rate (93.1% vs. 76.8%, *p* = 0.027; Fig. 3B).

**Fig. 4:**
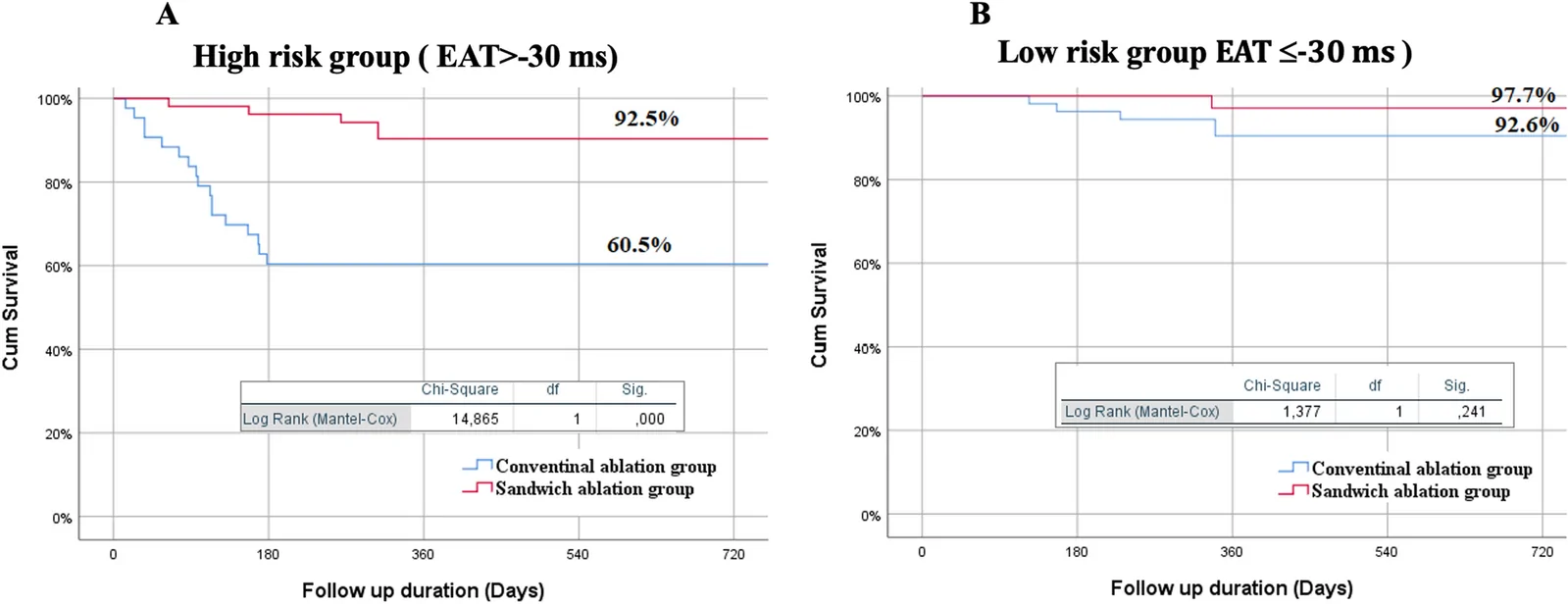
Recurrence-Free Survival Stratified by Activation Time Cut-off (–30 ms). Kaplan-Meier analysis stratified by activation timing. In the high-risk group (EAT >–30 ms), sandwich ablation significantly improved recurrence-free survival compared to conventional ablation (92.5% vs. 60.5%; *p* < 0.001). In the low-risk group (EAT ≤ − 30 ms), the difference was not statistically significant (97.7% vs. 92.6%; *p* = 0.241). EAT = earliest activation time

Subgroup analyses integrating anatomical origin and activation timing revealed that the clinical benefit of sandwich ablation was primarily observed in high-risk patients ( EAT > − 30 ms). In this high-risk group, sandwich ablation significantly improved recurrence-free survival compared to conventional ablation (92.5% vs. 60.5%, log-rank *p* < 0.001; Fig. 4A). In contrast, among patients with Low risk (EAT ≤ − 30 ms), no statistically significant difference was found between the groups (97.7% vs. 92.6%, *p* = 0.241; Fig. 4B).

**Fig. 5:**
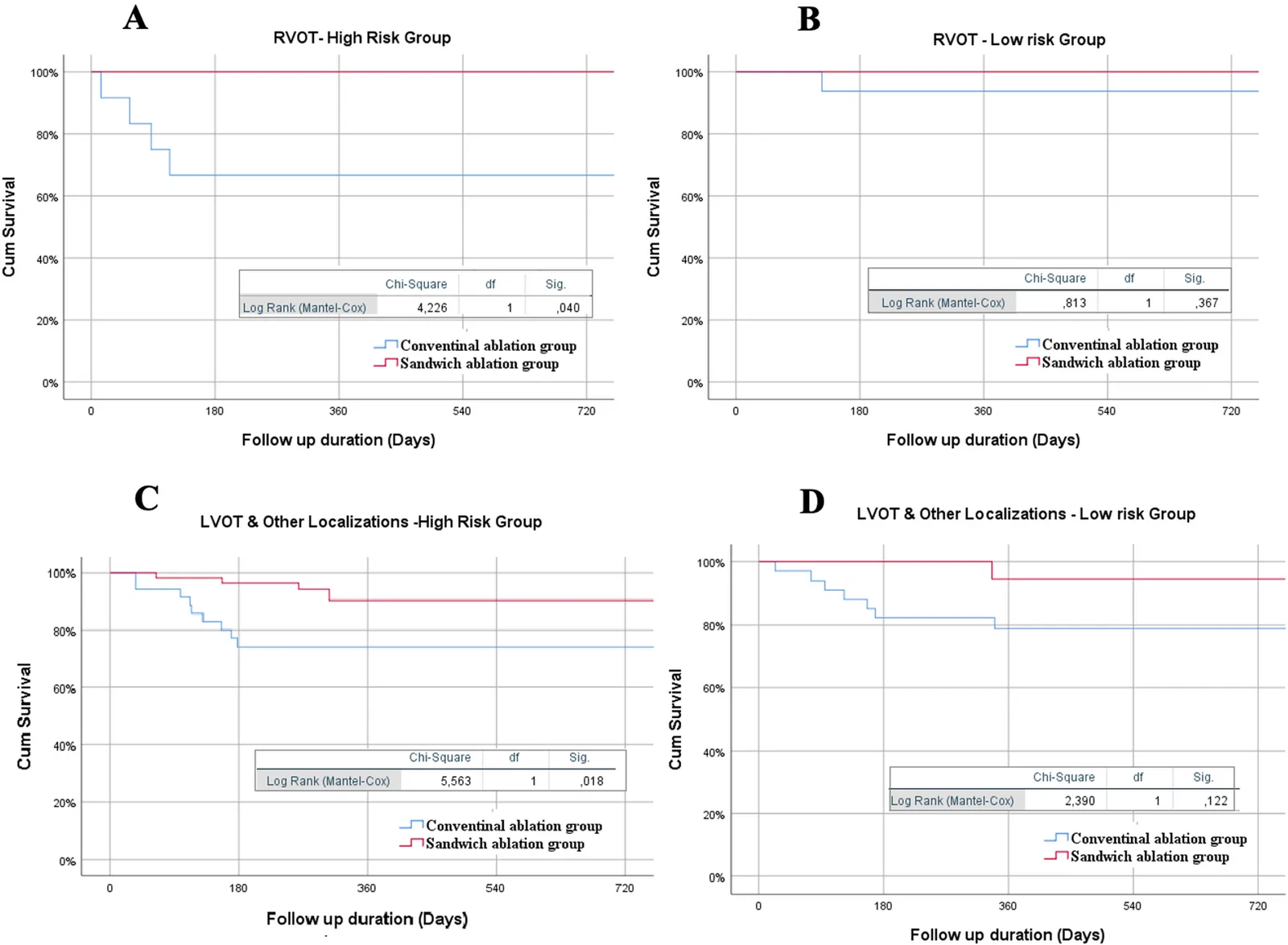
Subgroup survival analysis by anatomical location and risk level Kaplan-Meier curves illustrating recurrence-free survival stratified by both anatomical location and earliest activation risk category. Sandwich ablation significantly improved outcomes in:- RVOT-high risk: 100% vs. 72.1% (*p* = 0.040)- LVOT & other-high risk: 90.6% vs. 68.0% (*p* = 0.018) No significant difference was observed in low-risk groups (RVOT and LVOT & others; *p* > 0.05)

Further stratification by anatomic location and activation-based risk level confirmed this trend:

- RVOT with EAT > − 30 ms: 100% vs. 72.1%, *p* = 0.040 (Fig. 5A).

- RVOT with EAT ≤ − 30 ms: 100% vs. 95.2%, *p* = 0.367 (Fig. 5B).
- LVOT & other locations with EAT >–30 ms: 90.6% vs. 68.0%, *p* = 0.018 (Fig. 5C).
- LVOT & others with EAT ≤ − 30 ms: 96.8% vs. 86.3%, *p* = 0.122 (Fig. 5D).

## Discussion

In this propensity-matched cohort study, we demonstrated that a systematic anatomy-guided dual-sided ablation strategy—termed the “sandwich” approach—significantly improved recurrence-free survival in patients undergoing ablation for idiopathic PVCs, particularly among those with earliest activation timing (>–30 ms). Importantly, this benefit was achieved without increasing the risk of procedural complications, suggesting that the sandwich strategy may offer a safe and effective option to enhance lesion transmurality and long-term outcomes in anatomically challenging arrhythmic substrates.

Our findings are consistent with and expand upon previous literature emphasizing the anatomical complexity and electrophysiological inaccessibility of arrhythmias originating from the LVS. The review by Kuniewicz et al. clearly highlights the structural division of the LVS into accessible and inaccessible zones, shaped by the surrounding vasculature and epicardial fat, which significantly complicates ablation attempts in this region [3]. In line with this anatomical framework, our study demonstrates that a dual-sided approach may overcome these limitations by targeting both the endocardial and adjacent epicardial aspects of the arrhythmogenic substrate, thereby increasing the likelihood of transmural lesion formation.

Notably, the ISESHIMA-SUMMIT study recently investigated anatomical vs. electrophysiological strategies in LVS arrhythmias and found that successful ablation from adjacent endocardial sites (endocardial closest site or endo- earliest activation site) was closely linked to specific anatomical proximity metrics, such as the epi- earliest activation site to endocardial closest site distance and timing interval [12]. While their approach remained primarily unidirectional (endo or epi), our study complements these findings by emphasizing that simultaneous bidirectional ablation (“sandwich” strategy) can be particularly effective, especially in cases where conventional unipolar ablation fails due to intramural or epicardially shielded foci.

In a similar vein, Yang et al. reported that stepwise dual-sided ablation is often required in intramural ventricular arrhythmias originating from the basal inferior septum, due to ambiguous local activation times and poor pace-match from single-sided approaches [14]. Their work supports our assertion that certain PVC origins demand a multipolar, anatomical perspective to maximize efficacy, particularly when electrograms are non-specific or access is limited.

One of the most important contributions of our study is the identification of a clinically vulnerable subgroup characterized by early—but not enoughly premature—activation times, In these patients, the sandwich ablation strategy yielded significantly superior outcomes compared to conventional unipolar ablation (92.5% vs. 60.5% recurrence-free survival). This observation suggests that the arrhythmic focus in such cases may reside deeper within the myocardium or be insulated by epicardial adipose or fibrofatty tissue, limiting the effectiveness of ablation delivered from a single surface.

From a pathophysiological perspective, PVCs with less premature activation are often associated with functionally intramural origins, where both electrical insulation and anisotropic conduction hinder effective energy penetration. The presence of epicardial fat, especially in the left ventricular summit region, has been shown to reduce lesion depth and obscure electrogram clarity, particularly when accessed from the epicardial side. These features may necessitate bidirectional energy delivery—simultaneous or sequential—to achieve transmural lesion formation and durable arrhythmia suppression [9, 16]. Supporting this need for enhanced lesion sets, Wang et al. recently reported that delivering additional ablation lesions surrounding the index site significantly improved PVC-free survival in patients with outflow tract-PVCs, even without employing dual-sided ablation [9]. Their randomized study reinforces the idea that arrhythmogenic substrates may extend beyond the earliest activation site and require more extensive ablation to prevent recurrence. Our results build upon this concept by demonstrating that targeting anatomically opposite surfaces—beyond just adjacent tissue—can further improve outcomes, especially in intramural substrates.

Supporting this hypothesis, Watanabe et al. demonstrated that specific anatomical proximity metrics (e.g., epi- endocardial closest site to endocardial closest site distance) can predict ablation success in LVS arrhythmias when using single-sided approaches [12]. Similarly, Yang et al. reported that in basal intramural arrhythmias, neither endocardial nor coronary venous ablation alone was sufficient; instead, stepwise mirrored ablation was required to eliminate the arrhythmogenic substrat [14]. This concept is further supported by the findings of Ghannam et al., who showed that targeting anatomically adjacent myocardial or venous structures can be effective in intramural ventricular arrhythmias, even when these sites are not the earliest in activation timing [17]. While these prior approaches applied adjacent-site ablation selectively in specific scenarios, in our study the sandwich strategy was implemented as a predefined, systematic anatomy-guided approach following confirmation of acute success.

In contrast, patients with very early activation times (e.g., EAT ≤ − 40 or − 50 ms) likely have subendocardial or superficial epicardial foci, where conventional ablation techniques are often sufficient. In such cases, adding epicardial access or sandwich ablation may unnecessarily increase procedural complexity and risk—such as coronary injury or pericardial complications—without significant benefit.

Taken together, our findings support the use of EAT as a simple, objective, and physiologically meaningful stratification tool. A threshold around − 30 ms may help differentiate patients likely to benefit from anatomically enhanced approaches, such as the sandwich strategy, versus those amenable to standard single-sided ablation. This framework offers a practical, patient-tailored method for optimizing procedural strategy in idiopathic PVC ablation.

### Technical advantages and clinical utility of the sandwich strategy

The sandwich ablation strategy offers several technical and clinical advantages over conventional unipolar ablation, particularly in anatomically challenging or functionally intramural PVC substrates. By applying radiofrequency energy from both the endocardial and opposing surfaces, this approach increases the likelihood of effective transmural lesion formation—especially in areas like the LVS, where thick myocardial walls, epicardial fat, or poor catheter contact limit standard lesion delivery. From a biophysical standpoint, dual-sided energy application generates overlapping thermal fields, enhancing lesion depth while minimizing risks such as steam pops or impedance rise. This technique reduces the dependence on precise unipolar catheter positioning, which is often suboptimal in complex anatomical regions. More importantly, our data indicate that the greatest benefit is seen in patients with earliest activation times between > − 30 msn, a group that likely harbors deep intramural or epicardially shielded arrhythmogenic foci. In this high-risk subgroup, the sandwich strategy achieved markedly superior arrhythmia-free survival compared to standard ablation.

Therefore, beyond serving as a salvage technique, we propose the systematic use of sandwich ablation in patients with EAT > − 30 ms, where the anatomy and activation profile suggest that unipolar energy alone may be insufficient. In these cases, sandwich ablation should not be reserved as a backup, but rather considered an upfront strategy to improve procedural success and long-term outcomes.

### Study limitations

Despite its clinically meaningful findings, this study has several limitations. First, its retrospective, single-center design may introduce inherent biases, even though 1:1 propensity score matching was used to balance baseline characteristics. Second, the decision to apply sandwich ablation was operator-dependent, which may have led to variability in technique and lesion delivery. Third, while electroanatomic mapping protocols were standardized, differences in identifying the earliest activation site and annotating electrograms may have influenced EAT measurements and, consequently, subgroup classification. Furthermore, systematic mapping of adjacent venous or arterial structures using dedicated wires or small-caliber (e.g., 4-Fr) diagnostic catheters was not routinely performed, as the sandwich strategy was primarily anatomy-guided rather than mapping-driven, which limits direct electrophysiological confirmation of intramural substrate location. Coronary venous anatomy and its relationship to ablation sites were also not routinely assessed with advanced imaging modalities such as cardiac computed tomography or selective angiography. In addition, epicardial access was not employed, further limiting direct confirmation of intramural or epicardial substrate location.

Therefore, although our findings are hypothesis-generating and supported by mechanistic rationale, they require validation in prospective, multicenter, randomized clinical trials before broad clinical implementation.

## Conclusion

In this propensity-matched cohort study of patients undergoing ablation for idiopathic PVCs, we demonstrate that the sandwich ablation strategy significantly improves recurrence-free survival, particularly in those with near-threshold earliest activation times (EAT > − 30 ms). These findings suggest that a subset of patients harbors functionally intramural or anatomically insulated arrhythmic substrates that may not be adequately addressed with conventional unipolar energy delivery alone.

By integrating EAT as a procedural stratification tool, electrophysiologists may more accurately tailor ablation strategy to the underlying pathophysiology, enabling more effective lesion formation and reducing the likelihood of arrhythmia recurrence. The sandwich approach, which is safe, accessible, and technically feasible, offers a valuable addition to the contemporary arrhythmia ablation toolkit—particularly for anatomically challenging cases. Further prospective validation of this strategy and its integration into substrate-guided workflows may advance the personalization and success of idiopathic PVC ablation.

## Supplementary Information

Below is the link to the electronic supplementary material.

Supplementary Material 1

Supplementary Material 2

Supplementary Material 3

## Data Availability

No datasets were generated or analysed during the current study.
