# Supplementary material for: Planned anatomy-guided dual-sided (“Sandwich”) ablation improves recurrence-free survival in idiopathic premature ventricular contractions: a propensity-matched cohort study
Source: J Interv Card Electrophysiol. 2026 Feb 19;69(6):1333–44. doi: 10.1007/s10840-026-02252-7 (PMC13645921; doi:10.1007/s10840-026-02252-7)
Supplement: Supplementary file 1 [file 10840_2026_2252_MOESM1_ESM.docx]

# Supplemantary Table 1. Baseline Characteristics Before and After Propensity Score Matching

| Variable | Before Matching: Sandwich (n=97 pts) | Before Matching: Conventional (n=315 pts) | SMD (Before) | After Matching: Sandwich (n=97 pts) | After Matching: Conventional (n=97 pts) | SMD (After) |
| --- | --- | --- | --- | --- | --- | --- |
| Age, years | 50.9 ± 11.6 | 52.1 ± 12.2 | 0.10 | 50.9 ± 11.6 | 50.4 ± 10.9 | 0.04 |
| Male sex, n (%) | 51 (52.6%) | 161 (51.1%) | 0.03 | 51 (52.6%) | 50 (51.5%) | 0.02 |
| LVEF, % | 56.1 ± 4.7 | 55.8 ± 5.2 | 0.06 | 56.1 ± 4.7 | 56.3 ± 4.9 | 0.04 |
| QRS duration, ms | 108.3 ± 14.5 | 110.1 ± 15.2 | 0.12 | 108.3 ± 14.5 | 109.2 ± 13.7 | 0.06 |
| Baseline PVC burden, % | 22.8 ± 4.5 | 23.2 ± 4.8 | 0.09 | 22.8 ± 4.5 | 22.6 ± 4.2 | 0.04 |
| LVOT origin, n (%) | 39 (40.2%) | 128 (40.6%) | 0.01 | 39 (40.2%) | 41 (42.2%) | 0.04 |
| RVOT origin, n (%) | 44 (45.3%) | 130 (41.3%) | 0.08 | 44 (45.3%) | 45 (46.4%) | 0.02 |
