# Supplementary material for: Planned anatomy-guided dual-sided (“Sandwich”) ablation improves recurrence-free survival in idiopathic premature ventricular contractions: a propensity-matched cohort study
Source: J Interv Card Electrophysiol. 2026 Feb 19;69(6):1333–44. doi: 10.1007/s10840-026-02252-7 (PMC13645921; doi:10.1007/s10840-026-02252-7)
Supplement: Supplementary file 3 [file 10840_2026_2252_MOESM3_ESM.docx]

# Supplemantary Table 3. Comparison of Recurrence Rates between Sandwich and Conventional Ablation Strategies According to Earliest Activation Time Cut off -30 msn and Anatomic Localization

| Groups | Sandwich  Ablation Group n/N (%) | Conventional Ablation Group n/N (%) | p-value |
| --- | --- | --- | --- |
| Total EAT>-30 ms | 4/53 (7.5%) | 17/43 (39.5%) | 0.000 |
| RVOT EAT>-30 ms | 0/14 (0.0%) | 4/12 (33.3%) | 0.033 |
| LVOT+Other EAT>-30 ms | 4/39 (10.3%) | 13/31 (41.9%) | 0.004 |
| Total EAT ≤-30 ms | 1/44 (2.3%) | 4/54 (7.4%) | 0.375 |
| RVOT EAT ≤-30 ms | 0/11 (0.0%) | 1/16 (6.2%) | 1.000 |
| LVOT+Other EAT ≤-30 ms | 1/33 (3.0%) | 3/38 (7.9%) | 0.618 |

Abbreviations: EAT: Earliest Activation Time, LVOT: Left Ventricular Outflow Tract, RVOT: Right Ventricular Outflow Tract
